# THE INCREASING INVOLVEMENT OF AAAS IN ADDRESSING THE HOUSING NEEDS AND HOMELESSNESS OF OLDER ADULTS

**DOI:** 10.1093/geroni/igad104.0842

**Published:** 2023-12-21

**Authors:** Robert Graham, Isha Karmacharya, Traci Wilson, Molly French

**Affiliations:** Miami University, Oxford, Ohio, United States; Miami University, Oxford, Ohio, United States; USAging, Washington, District of Columbia, United States; USAging, Washington, District of Columbia, United States

## Abstract

While services addressing the housing needs and homelessness of older adults are not a core service of the OAA, the Aging Network is adapting to the levels of growing housing insecurity facing older adults. The need for affordable and accessible housing has increased in recent years. In the 2019 National Survey of AAAs, 30% of agencies reported a desire for training and technical assistance on better addressing housing/homelessness issues; by 2022 that interest increased to 46% of agencies. Further, 81% of AAAs said that they are now providing one or more housing/homelessness programs and services. AAA involvement in housing and homeless programs and services may range from providing home modifications, supportive housing, or even owning senior housing. Community partnerships play a critical role in meeting the housing needs of older adults. This presentation shows how AAAs have adapted to address these needs, including programs, services, community partnerships, and advocacy efforts.

